# Tumor cell lysate induces the immunosuppression and apoptosis of mouse immunocytes

**DOI:** 10.3892/mmr.2014.2606

**Published:** 2014-10-02

**Authors:** BOHAN DONG, GUANGLI DAI, LEI XU, YAO ZHANG, LIEFENG LING, LINGLING SUN, JUN LV

**Affiliations:** 1 Department of Biochemistry, Wannan Medical College, Wuhu, Anhui 241002, P.R. China; 2 Department of Gynaecology and Obstetrics, Traditional Chinese Medical Hospital of Wuhu, Wuhu, Anhui 241000, P.R. China

**Keywords:** tumor cell lysate, anti-tumor vaccine, immunosuppressive cells, immunocyte apoptosis, lung cancer, mouse immunocytes

## Abstract

Although tumor cell lysate (TCL) is a type of immunocyte stimulator, its immunosuppressive function must not be ignored. The present study reported that TCL prepared from a Lewis lung cancer cell was able to induce the development of immunosuppressive macrophages (MΦ) and tolerogenic dendritic cells. In addition, TCL upregulated the expression of CD69 in mouse splenocytes, and cell apoptosis and the percentage of regulatory T cells in mouse splenocytes simultaneously increased. Furthermore, the present study found that the immunosuppressive factor, hyaluronan, and the apoptosis inducers, Fas ligand and transforming growth factor-β, are present in TCL. These components may be associated with the emergence of immunosuppressive cells or splenocyte apoptosis. Thus, the present study has enriched our understanding of the composition of TCL and its negative regulatory effect on immunocytes.

## Introduction

Tumor cell lysate (TCL) refers to the tumor lysate mixture that results following the artificial lysis of tumor cells. This mixture has multiple tumor antigens and thus possesses the ability to induce the activation of antitumor cells. However, TCL has a dual effect on immune cells. TCL not only activates these immune cells, but may also induce immunosuppression. For patients with myeloma that receive treatment with an autologous dendritic cell (DC) vaccine, DCs should be incubated with the TCL derived from myeloma *in vitro*. During this incubation, TCL may induce a subset of DCs with stimulatory activity, while having the ability to induce immunotolerance in another subset of DCs (1). However, a transfusion of this subset of DCs may attenuate the specific immune response of effector T cells to tumor-associated antigens, resulting in antigen-specific immunotolerance of T cells and subsequently a low tumor response rate and short survival time (1). In a previous study, a TCL derived from Lewis lung cancer cells, in combination with mycobacterial heat shock protein 65 (MHSP65), was used to prepare MHSP65-TCL, which was found to activate the immune cells of mice during the early phase of immunization and was characterized by an upregulation of CD69 expression. However, during the late phase, the immune cell response to the vaccine decreased, accompanied by a reduction in the expression levels of CD69 (2).

The dual effects of TCL are ascribed to the different characteristics of the lysate components. As a complex mixture, TCL contains not only antigens, but also other substances that inhibit the immune cells or even induce cell apoptosis. Hyaluronan (HA) is a component of the extracellular matrix and can be secreted by nine types of cancer cells, including human lung cancer 95D cells and liver cancer HepG2 cells. The HA secreted by these cells may induce the production of immunosuppressive monocyte/macrophages (MΦ) and DCs *in vitro* (3). Although these cells are in an active state, they may induce the apoptosis of T lymphocytes, while they, themselves, are also susceptible to apoptosis (4). In addition to the inhibitory effect on immune cells, certain proteins secreted by cancer cells may directly induce cell apoptosis. Fas ligand (Fas-L) and transforming growth factor-β (TGF-β) are proteins that are closely associated with the apoptosis of immune cells and are potentially localized in the TCL. Fas-L can bind to Fas on immune cells to induce the activation of caspase in immune cells and to further induce cell apoptosis (5). However, TGF-β may act on the TGF-β receptor to activate the extracellular signal-regulated kinase/mitogen-activated protein kinase signaling pathway resulting in the apoptosis of immune cells (6). In addition, immunohistochemistry and western blotting demonstrated that the two proteins were expressed in the cancer cells. Western blot analysis demonstrated that Fas-L is expressed in 16 human lung cancer cell lines. In addition, immunohistochemistry results have demonstrated the expression of Fas-L in 23 out of 28 types of resected lung cancer (7). Furthermore, breast cancer cells also express Fas-L and lymphocyte apoptosis has been observed in adjacent normal tissues surrounding breast cancer tissues (8). Immunohistochemistry results have also revealed that the expression of TGF-β was at a high level in 45 lung cancer samples (9). Patients with high TGF-β expression levels in lung cancer cells were found to have a significantly shorter survival time following surgery (10). Immunohistochemistry and western blot assays enable the detection of intracellular proteins and thus, it was hypothesized that the TCL prepared from cancer cells may contain Fas-L and TGF-β.

On the basis of the aforementioned findings, the present study was undertaken to determine the concentration of HA, pro-apoptotic Fas-L and TGF-β in the TCL from Lewis cells, and to further investigate whether TCL induces the production of immunosuppressive cells and the apoptosis of immune cells through these proteins.

## Materials and methods

### Mice and cell lines

Female C57BL/6 mice were purchased from Nanjing Qinglong Mountain Laboratory Animal Co., Ltd. (Nanjing, China) and maintained in microisolator cages under pathogen-free conditions. All mice were studied at 6–8 weeks of age. Experimental manipulation of the mice was undertaken in accordance with the National Institute of Health Guide for the Care and Use of Laboratory Animals (Bethesda, MA, USA). A mouse Lewis lung cancer cell line was purchased from the American Type Culture Collection (Manassas, VA, USA) and maintained in high-glucose Dulbecco’s modified Eagle’s medium (Wuhan Boshide Biotechnology Co., Wuhan, China) supplemented with 10% fetal calf serum (FCS; Invitrogen Life Technologies, Carlsbad, CA, USA), 100 U/ml penicillin and 100 μg/ml streptomycin (Sigma-Aldrich, St. Louis, MO, USA). This study was approved by the Ethics Committee of Wannan Medical College (Wuhu, China).

### Preparation of TCL

To prepare the TCL, cultured Lewis cells were lysed using a freezing-thawing cycle in a 0.85% NaCl solution. This was repeated five times in rapid succession, between −70°C and 37°C and then refrozen and stored in a −70°C refrigerator until use. Each of the TCLs were detected under a microscope (Olympus Corporation, Tokyo, Japan) using trypan blue staining (Sigma-Aldrich, St. Louis, MO, USA) following the final thawing.

### Isolation of monocytes and culture of DCs

Peritoneal MΦs were isolated using plastic adhesion and further subset purification was performed with magnetic beads (Miltenyi Biotech, Bergisch, Gladbach, Germany) and specific biotin-conjugated antibodies (BD Biosciences, Franklin Lakes, NJ, USA), yielding >98% cell purity. Subsequently, MΦ (1×10^6^ cells/ml) were cultured in DMEM medium (Wuhan Boshide Biotechnology Co., Wuhan, China) with 10% FBS and added to either 0.85% NaCl or a TCL prepared from 1×10^6^ Lewis cells for 72 h. The culture supernatant was collected every 24 h. To prepare murine DCs, bone marrow cells were harvested from the tibiae and femurs of the C57/BL6 mice and depleted of red blood cells using a red blood cell lysis buffer (Sigma-Aldrich). Bone marrow cells were cultured in an RPMI-1640 medium containing 10% FBS, 100 U/ml penicillin, 100 μg/ml streptomycin and 50 μM 2-mercaptoethanol (Invitrogen Life Technologies), supplemented with 20 ng/ml murine granulocyte-macrophage colony-stimulating factor (GM-CSF) and IL-4 (Miltenyi Biotech) in the presence of NaCl or a TCL prepared from Lewis cells (1×10^6^). On days 3 and 6, the culture medium was replaced with a fresh medium supplemented with GM-CSF. From day 5, the culture supernatant was collected every 24 h.

### Flow cytometric analysis

The mouse spleen cells (1×10^6^/ml) that were co-cultured with either TCL prepared from Lewis lung cancer cells (1×10^6^) or 0.85% NaCl for 48 h were collected, washed and resuspended in phosphate buffered-saline (PBS) supplemented with 1% heat-inactivated fetal bovine serum. Thereafter, the mouse spleen cells were stained with Annexin V and propidium iodide (BD Pharmingen, San Diego, CA, USA) to detect apoptosis or stained with fluorescein isothiocyanate-labeled anti-CD4 and phycoerythrin-labeled anti-CD25 monoclonal antibodies (mAB; BD Pharmingen) to analyze the activation of regulatory T (Treg) cells. The cells were then put on ice in the dark for 30 min, washed with a fluorescence-activated cell sorting buffer (1X PBS) and analyzed by flow cytometry (FACS Calibur; Becton-Dickinson, San Jose, CA, USA).

### Western blot analysis

The TCL was prepared from Lewis cells (1×10^6^) and was then separated using SDS-PAGE. The protein was transferred on gel onto a nitrocellulose membrane. The membrane was incubated with an anti-mFas-L mAb (Wuhan Boshide Biotechnology Co.) and an anti-rabbit polyclonal IgG-horseradish peroxidase successively (Wuhan Boshide Biotechnology Co.). To detect the membrane protein, 0.01 g 3,3′-diaminobenzidine (Sigma) was dissolved in 10 ml of 1X PBS with 0.25 g NiSO_4_ and 15% H_2_O_2_ (1.5 μl).

### DNA ladder

The spleen cells (1×10^6^) were treated with TCL that was prepared from either Lewis lung cancer cells (1×10^6^) or 0.85% NaCl for 48 h and were then collected and washed twice with PBS. The splenocyte DNA was extracted using the Genomic DNA Mini Preparation kit with Spin Column (Beyotime Institute of Biotechnology, Haimen, China). Subsequently, ~15 μg DNA was loaded onto a 1.5% agarose gel and analyzed using electrophoresis. The gel was stained with ethidium bromide and visualized under ultraviolet light.

### ELISA

Concentrations of tumor necrosis factor-α (TNF-α), interleukin (IL)-10, HA and TGF-β were determined using ELISA kits (Wuhan Boshide Biotechnology Co.).

### Statistical analysis

In the present study, the data on cytokine concentrations and surface marker expression are presented as the mean ± standard deviation. Statistical significance was determined using Student’s t-test. P<0.05 was considered to indicate a statistically significant difference.

## Results

### HA in TCL from Lewis cells induces the production of immunosuppressive MΦ and DCs in mice

**Figure 1:**
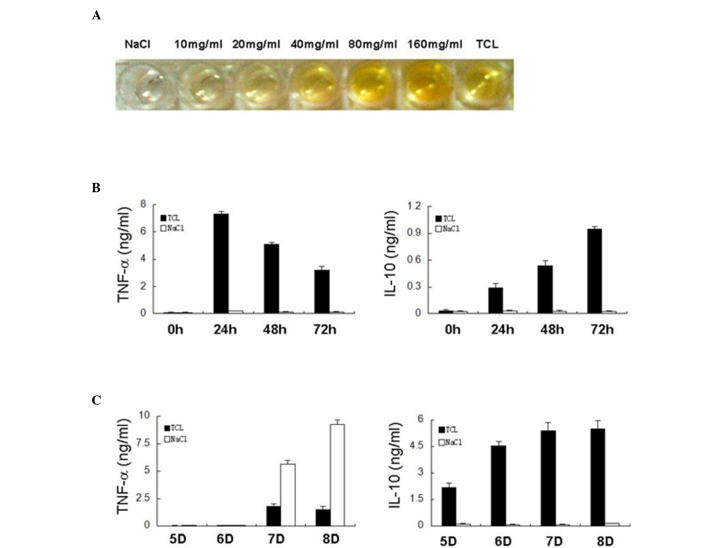
TCL induces the development of immunosuppressive macrophages and dendritic cells. (A) Lewis lung cancer cells (1×10^6^) were used to produce TCL. The HA in TCL was detected by ELISA and the concentration of HA was calculated by comparing the actual OD value of TCL to that of different HA standard samples. The actual OD value of TCL was: TCL (well 7) OD - NaCl (well 1) OD. The actual OD of the standard sample was: HA standard samples (wells 2–6) OD - Blank well OD. (B) Mouse monocytes were cultured for 3 days in a medium containing 0.85% NaCl (white bars) or with TCL from Lewis lung cancer cells (black bars). The production of the TNF-α and IL-10 cytokines was determined by ELISA. (C) Purified mouse DCs were cultured for 6 days with granulocyte-macrophage colony-stimulating factor and IL-4 in a medium containing 0.85% NaCl (white bars) or with TCL from Lewis lung cancer cells (black bars). Thereafter, the DCs were stimulated with lipopolysaccharide for 24 h and the production of the TNF-α and IL-10 cytokines was determined by ELISA. The data regarding cytokine production are presented as the mean ± standard deviation of three experiments. TLC, tumor cell lysate; HA, hyaluronan; OD, optical density; NaCl, sodium chloride; IL, interleukin; TNF-α, tumor necrosis factor-α.

HA is a matrix required for the growth of cancer cells. HA is secreted by cancer cells and may induce the production of immunosuppressive cells, resulting in the inability of immune cells to kill cancer cells (11–13). Certain cells can synthesize HA, including liver cancer HepG2 cells, cervical cancer HeLa cells and lung cancer 95D cells (3). To investigate whether HA is present in the TCL of mouse Lewis lung cancer cells, Lewis cells were subjected to repeated freezing and thawing to prepare the TCL. ELISA was then performed to detect HA in the TCL. The results demonstrated the presence of mouse-derived HA in the TCL. Following comparison of HA in the TCL with standard HA, the concentration of HA in the TCL was 42 mg/ml in the Lewis lung cancer cells (1×10^6^; Fig. 1A). In our previous study, the mice were immunized at four time points. Thus, the concentration of accumulated HA may be >42 mg/ml *in vivo* (2). HA may induce the production of immunosuppressive MΦs and DCs. Following the detection of HA in the TCL, whether TCL could induce the production of these immunosuppressive cells was further investigated. TCL from the Lewis cells was used to treat mouse MΦs and DCs independently and then the levels of TNF-α and IL-10 secreted by the MΦs and DCs were detected. As shown in Fig. 1B, after 24 h treatment with TCL, the concentrations of TNF-α and IL-10 secreted by the MΦs were markedly increased. The concentration of TNF-α from the MΦs began to decrease at 48 h. However, the concentration of IL-10 continuously increased and remained at a high level at 72 h. Overall, following incubation with the TCL, the mouse MΦs had an increased secretion of TNF-α during the early phase that continuously decreased thereafter. However, IL-10 secreted by the mouse MΦs continuously increased. Following NaCl treatment, the quantity of TNF-α and IL-10 from the MΦs remained stable at 24, 48 and 72 h. At four time points, the TNF-α concentration was 0.067, 0.174, 0.116 and 0.117 pg/ml, respectively (P<0.01 vs. TCL group). The IL-10 concentration was 0.022, 0.029, 0.025 and 0.023 pg/ml, respectively (P<0.01 vs. TCL group).

As with the mouse MΦs, the expression levels of IL-10 gradually increased in the immature mouse DCs after 6 days incubation with TCL *in vitro* (Fig. 1C). On day 5, the DCs remained immature and IL-10 secretion began to increase. When these cells had become completely mature on day 6, the IL-10 secretion continuously increased on days 7 and 8. By contrast, the secretion of IL-10 in the mouse DCs remained unaltered following NaCl treatment regardless of the maturation of the DCs. However, when NaCl-treated DCs had become mature, lipopolysaccharide (LPS) could stimulate the excessive secretion of TNF-α by mature DCs. Following a 24 (day 7) and 48 h (day 8) treatment with LPS, the TNF-α concentration was 5.655 and 9.255 ng/ml, respectively. In addition, the TCL-treated DCs were insensitive to LPS. After a 24 (day 7) and 48 h (day 8) treatment with LPS, the TNF-α concentration was 1.790 and 1.515 pg/ml, respectively (P<0.01 vs NaCl control).

### Fas-L and TGF-β in TCL from Lewis cells induces the apoptosis of mouse lymphocytes

**Figure 2:**
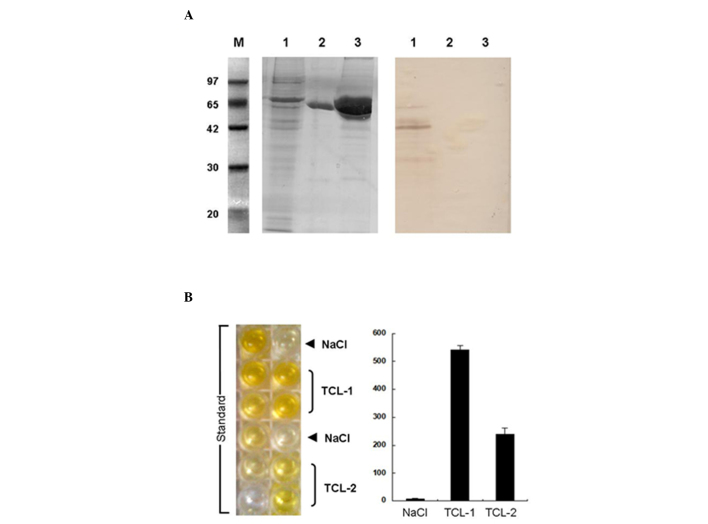
Fas-L and TGF-β expression in TCL. (A) TCL was prepared from 1×10^6^ Lewis lung cancer cells. Immunoblot analysis was processed to detect the Fas-L in TCL with anti-Fas-L antibody. FasL (Lane 1 of NC membrane) was detected in the TCL, but bovine serum albumin (Lane 2 of NC membrane) and mycobacterial heat shock protein 65 (Lane 3 of NC membrane) control proteins were not detected. (B) TCL was produced from 1×10^6^ or 2×10^6^ Lewis lung cancer cells. ELISA was processed to detect the TGF-β in TCL and the concentration of HA was calculated by comparing the actual OD value of TCL to that of different HA standard samples. The actual OD value of TCL was: TCL OD (TCL-1 or TCL-2 well) - NaCl OD. The actual OD of the standard sample was: HA standard samples OD - Blank well OD. TCL-1 indicates TCL from 2×10^6^ Lewis lung cancer cells. TCL-2 signifies TCL from 1×10^6^ Lewis lung cancer cells. NC, nitrocellulose; Fas-L, Fas ligand; TGF-β, transforming growth factor-β; TCL, tumor cell lysate; HA, hyaluronan; OD, optical density; NaCl, sodium chloride; M, marker.

Cancer cells may induce the apoptosis of immune cells by secreting pro-apoptotic factors (Fas-L and TGF-β), which is one of mechanisms underlying the escape of cancer cells from antitumor immunity (14–19). In the present study, western blot analysis and ELISA were performed to detect Fas-L and TGF-β in the TCL from Lewis lung cancer cells (Fig. 2A). Western blot analysis demonstrated that the Fas-L concentration was 200 ng/ml in the TCL from the Lewis cells (1×10^6^) and that the Fas-L detected by the western blot assay had a high specificity and could not react with non-specific proteins (including MHSP65 and bovine serum albumin). ELISA demonstrated that the TGF-β concentration was 239.64 pg/ml in the TCL from the Lewis cells (1×10^6^). However, no TGF-β was identified in the TCL from the Lewis cells in the NaCl group (Fig. 2B).

**Figure 3:**
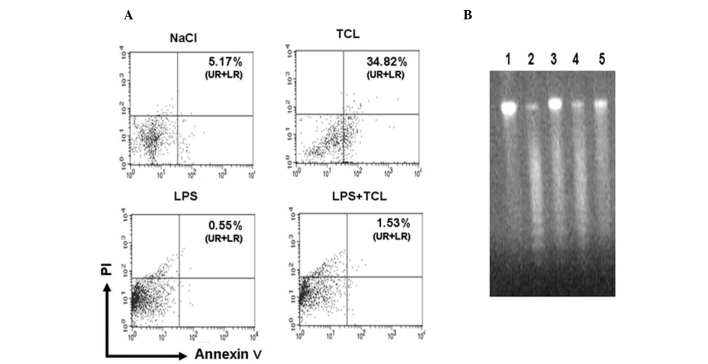
TCL induces the apoptosis of mouse splenocytes. Mouse splenocytes were cultured for 48 h in a medium containing 0.85% NaCl, TCL from Lewis lung cancer cells, MHSP65, LPS alone or LPS + TCL. The level of apoptosis in slpenocytes was determined by flow cytometry or DNA ladder. (A) The numbers in each plot image represent the positive percentage of Annexin V (UR percentage + LR percentage). Representative data from one of the three experiments are shown. (B) Genomic DNA of mouse splenocytes cultured with medium containing 0.85% NaCl (Lane 5), TCL from Lewis lung cancer cells (Lane 2), MHSP65 (Lane 4), LPS alone (Lane 1) or LPS + TCL (Lane 3) was analyzed by agar-gel. The ladder degraded bands of genome DNA indicate apoptosis of splenocytes. MHSP65, mycobacterial heat shock protein 65; LPS, lipopolysaccharide; TCL tumor cell lysate; PI, propidium iodide; NaCl, sodium chloride; UR, upper right; LR, lower right.

Subsequently, the mouse splenocytes were incubated with the TCL for 48 h *in vitro*. The results demonstrated that TCL was able to markedly stimulate the apoptosis of mouse splenocytes (Fig. 3A). When compared with the NaCl group, the apoptotic rate was as high as 34.82% (P<0.05). In addition, the apoptotic rate of the mouse splenocytes decreased to 0.55% following incubation with LPS for 48 h. If the mouse splenocytes were simultaneously treated with TCL and LPS, their apoptotic rate increased to 1.53% (P<0.05 vs. LPS group). A DNA ladder assay also revealed that TCL was able to promote the apoptosis of mouse splenocytes. In the TCL-treated mouse splenocytes, electrophoresis of the genomic DNA revealed evident DNA ladders. In the NaCl group and the LPS group, electrophoresis of the genomic DNA failed to show any DNA ladders. However, following simultaneous treatment with LPS and TCL, electrophoresis of the genomic DNA from the mouse splenocytes again demonstrated DNA ladders (Fig. 3B).

### TCL from Lewis cells upregulates CD69 expression in mouse splenic lymphocytes and induces production of Treg cells

**Figure 4:**
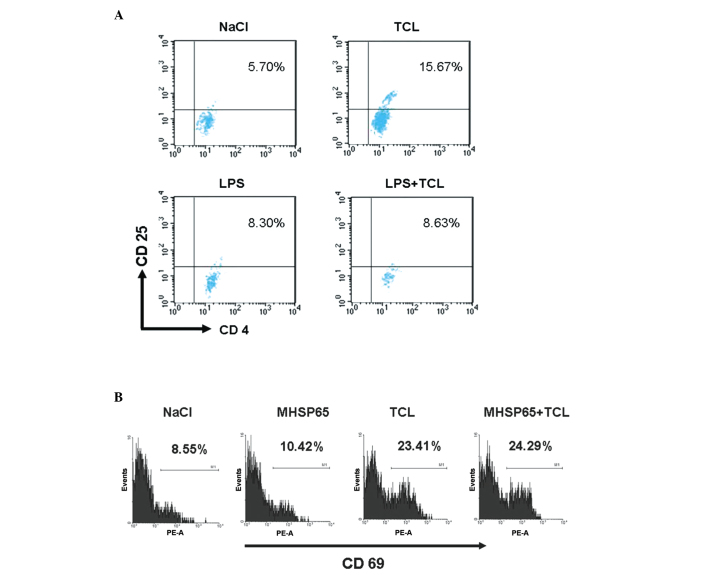
TCL upregulates the expression of CD69 and induces the formation of Tregs. (A) Mouse splenocytes were cultured for 48 h in medium containing 0.85% NaCl, TCL from Lewis lung cancer cells, LPS alone or LPS with TCL (LPS + TCL). The expression levels of CD4 and CD25 were determined by flow cytometry. The number in each upper right quadrant is the percentage of CD4/CD25 double-positive T cells. Representative data from one of the three experiments are shown. (B) Mouse splenocytes were cultured for 48 h in medium containing 0.85% NaCl, TCL from Lewis lung cancer cells, MHSP65 alone or MHSP65 with TCL (MHSP65 + TCL). The expression of CD69 was determined by flow cytometry. The number in each histogram represents the CD69 mean fluorescence intensity of the sample. Tregs, regulatory T cells; NaCl, sodium chloride; TCL, tumor cell lysate; MHSP65, mycobacterial heat shock protein 65; LPS, lipopolysaccharide.

The transformation of CD4^+^ T cells into Treg cells is an inducer of tolerance to tumor vaccination (20). In order to investigate whether TCL-induced tolerance to tumor vaccines is associated with Treg cells, mouse splenic lymphocytes were treated with TCL *in vitro* for 48 h and then flow cytometry was performed to detect the Treg cells (Fig. 4A). The results demonstrated that following TCL treatment, the proportion of Treg cells was 15.67% in the CD4^+^ T lymphocytes. However, in the NaCl group, the proportion of Treg cells was 5.70% (P<0.05). Notably, in the LPS-treated CD4^+^ T lymphocytes, the proportion of Treg cells was 8.30% and in the CD4^+^ T lymphocytes treated with LPS and TCL, the proportion of Treg cells was 8.63%, suggesting that the number of Treg cells was not significantly increased (P>0.05 vs. LPS group). Treg cells are a group of immunosuppressive CD4^+^ T lymphocytes. The production of Treg cells is associated with CD69 expression and TGF-β secretion in immune cells (21). Consistently, after 48 h treatment with TCL, the expression of CD69 was significantly increased in the mouse splenic lymphocytes with CD69 expressed in 23.41% of the splenic lymphocytes (Fig. 4B). However, in the NaCl group, CD69 expression was observed only in 8.55% of the splenic lymphocytes (P<0.01).

## Discussion

TCL has long been used as a stimulator of immune cells and as an antitumor vaccine. However, there are substances in TCL that can exert an immunosuppressive effect and induce apoptosis, resulting in adverse effects on the action of TCL. Thus, it is imperative to detect these substances and to investigate the mechanisms underlying the effect of these substances on TCL-mediated immunoactivation (22,23). In our previous studies, TCL was prepared from Lewis lung cancer cells and the results demonstrated that the use of TCL could induce antitumor immunity that was not maintained during the late phase (2). It was hypothesized that this may be attributed to certain components in the TCL that could inhibit immune cells. In the present study, the results demonstrated the presence of HA in the TCL from Lewis lung cancer cells and it has been confirmed that HA is closely associated with the production of immunosuppressive immune cells (MΦ and DC). The concentration of HA was 42 mg/ml in the TCL from the Lewis lung cancer cells (1×10^6^). In prior animal studies, the animals were immunized at several time points and thus, the *in vivo* HA concentration may be higher. At this concentration, HA was able to induce the production of immunosuppressive cells (24). The TCL-treated MΦs were consistently found to possess an elevated capability to secrete TNF-α and the secretion of TNF-α decreased over time. However, the secretion of IL-10 gradually and continuously increased. In the NaCl-treated MΦs, the secretion of TNF-α and IL-10 demonstrated an opposite tendency to that of the TCL-treated MΦs. Similar findings in the secretion of TNF-α and IL-10 by mouse DCs were also demonstrated following TCL or NaCl treatment. These findings suggest that following TCL treatment, the MΦs and DCs transform into a special group of immunosuppressive MΦs. The immunosuppressive MΦs and DCs are characterized by a reduction in the secretion of TNF-α and IL-12 and an increase in the secretion of IL-10, an inhibitory cytokine (25). These immunosuppressive MΦs and DCs may be derived from immature mouse MΦs and DCs following incubation with the HA in the TCL. If immunosuppressive MΦs and DCs are present, they may secrete a large quantity of IL-10 to induce the apoptosis of T lymphocytes. In addition, immunosuppressive MΦs and DCs are also susceptible to a secondary apoptosis following transient activation, which may result in a reduction in the proportion of antigen-presenting cells in the immune cells and may affect TCL-activated antitumor immune cells (4,26).

In addition to HA, two pro-apoptotic factors (Fas-L and TGF-β) were also detected in the TCL. In the TCL from Lewis lung cancer cells (1×10^6^), the concentration of Fas-L and TGF-β was 200 ng/ml and 239.64 pg/ml, respectively. The two proteins may assist cancer cells in escaping antitumor immunity. Fas-L may bind to Fas on the immune cells to trigger a cysteine protease cascade to induce the apoptosis of antitumor immune cells and thus, these immune cells fail to exert an antitumor effect (27). TGF-β is a multifunctional cytokine that can be secreted by cancer cells and at the same time, act on themselves by exerting an antitumor effect. If there is TGF-β in the TCL, the TGF-β may act on the immune cells to inhibit their function or even induce the apoptosis of these immune cells. Thus, TGF-β assists cancer cells in evading the antitumor immunity and promotes cancer formation (28). In the present study, TCL was directly used to treat mouse splenocytes over 48 h and the apoptosis of these cells was observed (apoptotic rate: 34.82%). This may be attributed to the direct contact between the splenocytes and Fas-L/TGF-β in the TCL. The ability of TCL to induce cell apoptosis is potent and, as a potent immune activator, LPS fails to completely inhibit this effect. Thus, although there are cancer antigens in the TCL, which could activate immune cells as LPS does, other factors, including Fas-L and TGF-β, may also induce the apoptosis of immune cells (apoptotic rate of mouse splenocytes: 34.82%). This undoubtedly affects TCL-mediated antitumor immunity.

The apoptosis of immune cells is associated with not only the pro-apoptotic factors, but also with the upregulation of the expression of CD69 on these cells (29). If the expression of CD69 on T lymphocytes and natural killer NK cells increases, the CD69 cells may activate secondary messengers and protein kinases, including Ca^2+^ and protein kinase C, which further activate signaling pathways resulting in increased secretion of TGF-β by the T lymphocytes and NK cells. TGF-β acts on immune cells to induce their apoptosis (30). In our previous study and in the present study, the results demonstrated that CD69 expression in the mouse splenic lymphocytes significantly increased following treatment with TCL and the proportion of CD69 positive cells was 23.41%. Of note, of the TCL-treated mouse splenic lymphocytes, CD4^+^/CD25^+^ Treg cells were detectable. Treg cells are a group of CD4^+^ T lymphocytes with immunosuppressive capability and can inhibit the activation of certain immune cells, including CD4^+^ cells, CD8^+^ T cells, NK cells and monocytes (31). In addition, the inhibitory activity of Treg cells is associated with the excessive expression of CD69 on them (32). In our previous study, CD69 was used as a marker for the activation of immune cells (21). Cancer antigens in the TCL could effectively activate immune cells that were characterized by an increase in the expression of CD69 on these cells (33). However, although CD69 may be used as a marker for immune cell activation, the expression of CD69 is not involved in the activation of immune cells and the occurrence of antitumor immunity (34). CD69^−/−^ mice were found to have a more potent antitumor capability (35). If an anti-CD69 antibody was used to inhibit CD69 expression, an antitumor effect was observed in cancer-bearing mice, suggesting the antitumor effect of the CD69 antibody (30). Thus, as for the detection of immune activators in the TCL, other cellular surface molecules, including HLA-DR and CD38, may be used (36). In addition, inhibition of the expression of CD69 assists in increasing the immunocompetence of the TCL.

Taken together, the results of the present study demonstrated that HA, Fas-L and TGF-β were present in the TCL from Lewis lung cancer cells, and may induce the production of immunosuppressive MΦs and DCs, or directly induce the apoptosis of immune cells. These molecules may significantly compromise the immunocompetence of TCL or cause a non-response to TCL. In addition, TCL may activate CD69 expression, leading to the production of Treg cells, which may further increase immune cell inhibition and apoptosis, and thus further reduce the antitumor activity of the TCL. On the basis of the abovementioned findings, removal of HA, Fas-L and TGF-β from the TCL preparation and inhibition of CD69 expression may improve the antitumor activity of TCL.
